# Gender-related factors influencing tuberculosis control in shantytowns: a qualitative study

**DOI:** 10.1186/1471-2458-10-381

**Published:** 2010-06-29

**Authors:** Dami A Onifade, Angela M Bayer, Rosario Montoya, Marie Haro, Jessica Alva, Jessica Franco, Rosario Sosa, Betty Valiente, Enit Valera, Carolyn M Ford, Colleen D Acosta, Carlton A Evans

**Affiliations:** 1 Asociación Benéfica PRISMA, Carlos Gonzales 251 Maranga, San Miguel, Lima, Peru; 2 Wellcome Centre for Clinical Tropical Medicine & Dept of Infectious Diseases & Immunity, Imperial College London, Hammersmith Hospital Campus, W12 0NN, UK; 3 Laboratorio de Investigación y Desarrollo, Departamento de Microbiologia, Universidad Peruana Cayetano Heredia Facultad de Ciencias y Filosofía, Av. Honorio Delgado #430 - Urb. Ingeniería, San Martin de Porres, Lima, Peru

## Abstract

**Background:**

There is evidence that female gender is associated with reduced likelihood of tuberculosis diagnosis and successful treatment. This study aimed to characterize gender-related barriers to tuberculosis control in Peruvian shantytowns.

**Methods:**

We investigated attitudes and experiences relating gender to tuberculosis using the grounded theory approach to describe beliefs amongst key tuberculosis control stakeholders. These issues were explored in 22 semi-structured interviews and in four focus group discussions with 26 tuberculosis patients and 17 healthcare workers.

**Results:**

We found that the tuberculosis program was perceived not to be gender discriminatory and provided equal tuberculosis diagnostic and treatment care to men and women. This contrasted with stereotypical gender roles in the broader community context and a commonly expressed belief amongst patients and healthcare workers that female health inherently has a lower priority than male health. This belief was principally associated with men's predominant role in the household economy and limited employment for women in this setting. Women were also generally reported to experience the adverse psychosocial and economic consequences of tuberculosis diagnosis more than men.

**Conclusions:**

There was a common perception that women's tuberculosis care was of secondary importance to that of men. This reflected societal gender values and occurred despite apparent gender equality in care provision. The greatest opportunities for improving women's access to tuberculosis care appear to be in improving social, political and economic structures, more than tuberculosis program modification.

## Background

Tuberculosis is one of the most common infectious causes of death worldwide [1]. In 2007, the estimated global incidence was 139 cases per 100 000 population (a total of 9.3 million cases) [2] with a mortality rate of 27 deaths per 100 000 population (a total of 1.8 million deaths attributed to tuberculosis) [2]. Peru has an acclaimed Tuberculosis Control Program [3], available at no direct cost to all members of the population throughout the health service network. Its wide-ranging services consist of diagnostic tests (except some radiological tests), treatment and a social care component, which provides food packages and assistance with the cost of clinical consultations and supplementary tests, such as HIV testing. Directly Observed Treatment, Short Course (DOTS) was implemented nationally in 1991. Peru has made progress towards the Millennium Development Goals for tuberculosis, with 100% DOTS coverage and a reduction in the incidence of tuberculosis from 65 000 cases (265/100 000) in 1997 [1] to 44 815 cases (162/100 000) in 2006 [4]. However, the disease burden remains high and multi-drug resistant tuberculosis (MDTR-TB) incidence is increasing rapidly, which is responsible for 7.7% of tuberculosis cases in Peru [5].

Globally, tuberculosis kills more women than any other single infectious disease [6,7] and is strongly associated with poverty, with 95% of all tuberculosis cases occurring in the developing world [6]. Seventy percent of the world's poor are female and these women face the greatest obstacle to seeking healthcare and obtaining successful tuberculosis treatment [6]. A recent World Health Organization report on global tuberculosis control reported 1.8 male cases of tuberculosis disease for every female case [4]. This has been explained both by socio-cultural factors, since men have more social contacts and thus run a greater risk of exposure to contagious cases, [6] and by immunological differences between men and women that make males more susceptible than females to some infections [8-10]. These hypotheses have been questioned by other studies that found under-notification and hence under-treatment of female tuberculosis cases by passive case finding when compared with active case finding [11-13]. In some cultural contexts, women are financially dependent on men and cultural gender roles result in a lack of autonomy. This has been found to reduce women's access to healthcare, interaction with healthcare services and adherence to tuberculosis treatment [6,12,14]. Stigma, fear and the psychological suffering associated with tuberculosis are also important in determining health-seeking behaviour and are found to disproportionately affect women [7,12,14]. These findings have implications for the health of women, their status in society and the health of their children.

This qualitative study aimed to characterize gender-related barriers to tuberculosis control in Peruvian shantytowns, using semi-structured interviews and focus group discussions with tuberculosis patients and healthcare workers. Our literature search did not identify any published studies exploring this issue in Latin America. Furthermore, investigation of the interaction between gender and tuberculosis is warranted in Peru because there is a high tuberculosis burden and national statistics report that women are diagnosed with tuberculosis less often than men [4].

## Methods

The study was conducted in Ventanilla, which is located to the north of Lima, Peru. Ventanilla is made up of a series of shantytowns and covers a 70 000 km^2 ^area. It has a population of approximately 500,000 people distributed between 16 zones, each of which has a health establishment (15 health posts and one hospital) where the Ministry of Health operates the National Tuberculosis Control Program. Previous research in Ventanilla revealed an annual tuberculosis incidence rate of 242/100,000 in 2005 and an average daily income of 0.9 USD per person in tuberculosis affected families (unpublished data, CA Evans).

### Study population

The populations sampled were tuberculosis patients and healthcare workers who were purposively sampled to select informants who were able to provide in-depth, culturally-specific knowledge concerning tuberculosis and gender [15]. Patients were identified from the Tuberculosis Control Program register in Ventanilla. Inclusion criteria for patients were: those greater than 15 years of age who had a diagnosis of pulmonary tuberculosis within the last 3 years. We postulated that those over the age of 15 would be better able to provide culturally specific insight into gender roles and how this impacts upon tuberculosis within their community. We did not study children because they may not yet have developed the expectation to conform to culturally determined, stereotyped gender roles. Patients were stratified into groups by residential zone and sex, and were then randomly assigned to participate in interviews or focus group discussions.

Clinic healthcare workers involved in tuberculosis care were purposively sampled from the corresponding zonal health establishment and selected to represent a range of healthcare professionals whose sex ratio approximately reflected the sex ratio of tuberculosis care provision. Additionally, purposive sampling was used to recruit community healthcare workers involved in tuberculosis care within Ventanilla.

Table 1 shows the characteristics of the 43 participants recruited to the study. The sample size was determined by point of saturation (when the number of participants was large enough to provide sufficient information for no new evidence to emerge, [16] and small enough that the data could be fully analysed, given the richness of qualitative data).

**Table 1:** Characteristics of study participants.

*Type of participant*	*Number N = 43*	*Age [mean(range)] years*	*Zones represented*	*Occupations*
**Interviews (n = 22)**				
Male patients	6	34 (17-69)	4	1 unemployed, 5 informal irregular work
Female patients	8	34 (17-52)	7	6 unemployed, 1 cook, 1 entrepreneur
Healthcare workers (2 male, 6 female)	8	-	8	1 Clinic psychologist, 3 Clinic technical assistant, 3 nurses, 1 Clinic doctor
				
**Focus group discussions (n = 4)**				
Male patients	6	27 (18-31)	2	-
Female patients	6	31 (19-52)	6	-
Clinic healthcare workers (all female)	3	-	3	3 nurses
Community-bases healthcare workers (all female)	6	-	16 (regional workers)	3 health promoters, 2 nurses & 1 midwife

No interview participants were included in the focus group discussions.

Information regarding the age of healthcare workers was not recorded in order to ensure anonymity.

### Data collection methods

Accounts of the health experiences of male and female tuberculosis patients and healthcare workers were obtained by two methods of data collection: semi-structured interviews and focus group discussions.

#### Semi-structured interviews

We conducted 22 semi-structured interviews in which we explored: gender roles in the family and the importance of women's health; experienced and perceived gender barriers to accessing tuberculosis care and treatment adherence; and perceptions of the disease and associated stigma. Interviews were conducted in Spanish by the lead investigator (English origin) and another member of the research team (Peruvian origin) and lasted 60 to 90 minutes.

#### Focus Groups

We undertook four focus group discussions with male patients, female patients, clinic healthcare workers and community healthcare workers, respectively. Three case scenarios based on real-life tuberculosis patient experiences were discussed with each group. Issues relating to gender roles in the family, gender-specific interactions with tuberculosis diagnosis, treatment and the illness experience were also explored. Patients and healthcare workers were encouraged to provide what they thought to be the opinion in the general population and share their own experiences of tuberculosis disease. A Peruvian nurse facilitated all focus group discussions.

Issues explored in the interviews and focus groups were developed with a topic guide, which was improved after an initial pilot with seven community healthcare workers. The guide was semi-structured and flexible so that emergent themes could be included and discussed in subsequent data collection activities. Interviews and focus group discussions were conducted in Spanish, recorded using a digital recorder or by taking detailed notes, depending on the participant's preference. Participation in the study was voluntary and written informed consent was obtained. Anonymity was assured and no identifying information was collected. Ethical approval was obtained by Imperial College London, UK and Asociación Benéfica PRISMA (the non-governmental organization that coordinated this study and has an internationally accredited ethical committee in Lima, Peru).

### Data analysis

**Figure 1:**
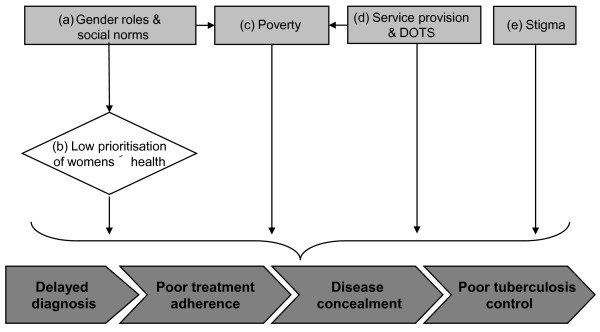
**Conceptual framework of the themes contributing to gender inequity in tuberculosis control in this study**.

Data from the semi-structured interviews and focus groups were transcribed and then translated from Spanish to English by an independent translator. Data was analysed using the grounded theory approach [16]. First, a set of transcripts was reviewed to identify possible concepts and categories. Next, a set of codes was developed based on these categories and all transcripts were subsequently coded. During the coding process, constant comparison was used to compare and review accounts given by male and female patients and by patients and healthcare workers. This iterative approach to data analysis was done in order to ensure that the most appropriate categories and themes had been formulated and that all perspectives were included. This method of analysis allowed emergent central messages or "themes" to be inductively derived from the statements made by the tuberculosis patients and healthcare workers [16,17], as illustrated in Figure 1 and characterized in more detail in Table 2.

**Table 2:** Concepts, categories and emerging themes in this study.

*Concepts*	*Categories*	*Emerging Themes*
**Gender roles in the family and their impact on women's health**		
	
Stereotyped roles Gender prejudice Cultural and social norms	1. Gender roles	(a) Gender roles
	
Men's health is a greater priority Children are a priority Self sacrifice	2. Low priority of women's health	(b) Low prioritization of women's health
Limited work opportunities for women Women are not financially independent	3. Low investment in women's health and nutrition	(c) Poverty
		
**Gender-related factors influencing tuberculosis access and care**		(d) Service provision and DOTS
Health beliefs Motivation Acceptance of illness	4. Awareness of tuberculosis related healthcare services	(e) Tuberculosis-related stigma
Knowledge of services Women involvement in community activities Limited economic resources TB care is free	5. Tuberculosis test-seeking behaviour	
DOTS and childcare DOTS and employment Inflexible health establishment opening hours	6. Tuberculosis treatment experiences	
Tuberculosis sufferers are perceived negatively in the community Concealment of diagnosis Lack of support Rejection Physical appearance	7. Tuberculosis-related stigma	
Healthcare workers attitude Scare-mongering Service is non-discriminatory Lack of structural amenities DOTS is insensitive	8. Perceptions of tuberculosis services and personnel	

## Results

This research identified categories (numbered 1-8) of gender-related factors that were found to impact (A) the health of women and (B) access to TB care (Table 2). These eight categories are presented with verbatim quotes from participants. Each quote is followed in brackets by a description of the participant. Patients are described by their age, sex and participation status in either an interview or focus group discussion. Healthcare workers are described by their job title, sex and participation status in either an interview or focus group discussion. The age of healthcare workers is not given to protect their anonymity. Verbatim translations are followed, when necessary, by text clarifying the contextually specific interpretation. Five themes that were derived from these eight categories and found to characterize gender-related barriers to tuberculosis control were: (a) gender roles; (b) low prioritization of women's health; (c) poverty; (d) service provision and DOTS; and (e) tuberculosis related stigma. The relationship between the eight categories identified and the five inductive themes are presented in Table 2.

### A. Gender roles in the family and their impact on women's health

#### 1. Gender roles

It was universally expressed by participants that gender prejudice was common in the shantytown community of Ventanilla. Women and men reported stereotypical gender roles in accordance with cultural and social norms. Women remained at home to care for the family whilst men provided economic sustenance for the family. This was reinforced by limited work opportunities for women, who were therefore financially dependent on their husbands.

*The woman has to take the emotional and economic burdens of the entire family. She has to take responsibility for the children and everything. It is the duty of the husband, if he is a good man, to bring money home*.

(Clinic nurse, woman, focus group discussion)

*There isn't so much work for women, so she stays home and has to depend on him (the spouse)*.

(39 years old, woman, interview)

#### 2. Low priority of women's health

Women's health was found to be a low priority in families, as is shown below in the perceived importance of men's compared to women's health, and how often healthcare was solicited for ill men compared to women.

Participants reported that gender prejudice extended into families' healthcare prioritization, with women reporting that their health had lower priority than that of the men in the family.

*I quarrel with my husband because I feel that he does not worry about my health*.

(30 years old, woman, interview)

*There are some cases where the men do not want the women to go to the health post. They oppose it because they have to spend (money). The man is not interested in the woman's health. He does not support her*.

(Clinic technical assistant, woman, interview)

Men affirmed that generally the health status of women was not given equal importance to that of men. A male patient illustrated this by recounting the relationship between his parents.

*In the case of my dad, when my mother was ill, my dad didn't ask how she felt, he didn't take her to the health post...but when he was ill for any pain at all, he would go straight to the post and wouldn't be concerned about the expenses... but when my mom felt bad, he didn't ask her anything, didn't even buy her a pill. He just took care of himself*.

(27 years old, man, focus group discussion)

Qualitative accounts suggest that women need permission from partners and other family members to access healthcare. One female tuberculosis patient reported that her partner actively prevented her from accessing healthcare services.

*My husband said, "you will be better soon without needing medical care"...so he did not give me money to go to the health centre*.

(30 years old, woman, interview)

This was in contrast to men for whom healthcare was often solicited on their behalf by their mothers, wives and other female relatives. Women would frequently seek information on behalf of their male relatives at the health post.

*Here again we can see the figure of the maternalistic woman, as if the husband were another child whom she must carry to consult a clinic doctor... We have the case of a man in Ventanilla whose wife solicited a sputum sample container for him. She went to collect his results and then his treatment because he said he couldn't because he was working*.

(Clinic nurse, woman, focus group discussion)

Distinctively, women mentioned concern for transmission to their children - not concern for their own health - as motivation for seeking medical advice. This again demonstrates the low priority of women's health.

*I went to the tuberculosis control program in the health post because I wanted to be cured. I did not want to contaminate my kids by leaving them exposed to my untreated tuberculosis*.

(34 years old, woman, interview)

#### 3. Low investment in women's health and nutrition

Participants described how low prioritization of women's health leads to low economic investment in women's healthcare by families.

*In some cases, women depend on their husbands and so need their consent to use health services. The husband does not want to spend his money, so he sends her to social services*.

(49 years old, woman, interview)

A patient illustrated this with an example of when her husband was ill and money was scarce, she was resourceful in finding the money for his tests and treatment. However, this was not reciprocated when she was ill.

*I used to go to his work to say he was sick and that I needed an advance. In that case, he told me "go, run, go to my work and tell them I am sick, that they should give you money." When I was sick, he said he could not help me and that I should go to borrow money from my sister to pay for the required tests*.

(39 years old, woman, interview)

Low investment in women's health also influenced their nutrition. Women reported that they compromised their own nutrition and potentially their ability to fight tuberculosis in order to ensure that other family members were adequately nourished. Some even used the food packages provided by the tuberculosis program, which are intended for patients' nourishment whilst on tuberculosis treatment, to feed their families.

*I prefer that my children eat. If there is little food in the night, I prefer they eat...My husband eats twice what I eat*... *I worried that my kids ate well, and I neglected myself...for that reason the illness advanced, became worse*.

(39 years old, woman, interview)

*Many times we have bad customs... Men recommend that the woman give the best portion to the husband and many times women do not eat. There is a custom that if the man works, he has to eat well...One must give him a complete dish with a good portion and the woman remains without eating... It happens because most of the time the man provides the money. He should eat well because he works and the woman gets sick*.

(Clinic nurse, woman, focus group discussion)

*The Tuberculosis Control Program simply gives us rice, tuna and kidney beans. Before they used to give us powdered milk and flour; now they do not. I want them to at least help us, with something more, (because) it is for the children*.

(39 years old, woman, interview)

### B. Gender-related factors influencing tuberculosis access and care

#### 4. Awareness of tuberculosis related healthcare services

Awareness of available tuberculosis-related health services was strikingly variable and greater for women than men. Primary sources of information included community health promotion initiatives, which were more commonly attended by women.

*The women participate in the community, in the "mothers' clubs" (a community-based support group for mothers with school-aged children), "glass of milk" (a government-subsidized program that provides food for school-aged children) and "popular kitchens" (government-subsidized program that provides cooked meals for people in poverty). When she is sick, she can comment on this there and receive her diagnosis. It can be faster for women*.

(Clinic nurse, woman, focus group discussion)

Women were also reported to attend the health establishment more frequently than men (who were usually at work during the day) in order to seek healthcare on behalf of their children and relatives. As a result, they had a higher chance of receiving health promotion information concerning tuberculosis in the form of leaflets, posters and educational talks by personnel at the health establishment. Clinic staff reported that although women were frequent visitors to the health establishment, they were often there to access services for family members, not for themselves.

*The fact that one goes to the health post and sees a quantity of women, it is exactly because the woman is in the house and she has time to go to the health post for the care of her children that have for example diarrhoea or respiratory infections. Very few women go to the health post for themselves... You see a lot of women there, but they do so for the children or because they are caring for their husband or a relative or neighbour*.

(Clinic nurse, woman, focus group discussion)

#### 5. Tuberculosis test-seeking behaviour

As reported in the previous section, women's lack of financial independence and the low prioritization of their health by family members acted as barriers to accessing healthcare. This also affected tuberculosis test-seeking by women, despite greater knowledge about tuberculosis care when compared to men. Participants reported that although tuberculosis care is free, the patient pays the cost of the initial consultation at the clinic and all analyses prior to the sputum test. In addition there may be hidden or indirect costs, such as the cost of a chest radiograph if the sputum test is equivocal, the expense of lost income whilst unable to work and the cost of travel to the health establishment. Participants reported that the cost of healthcare acted as a deterrent to seeking healthcare services.

*The analysis and X-rays were difficult, due to the economic situation*.

(24 years old, woman, interview)

*Women have more time, but sometimes do not go to the clinic doctor because of lack of money*.

(34 years old, woman, interview)

Limited work opportunities for women in Ventanilla left the majority of women dependent on their spouses, family or social services. Most women reported that they had difficulty meeting the cost of healthcare, and this contributed to delays in test-seeking.

*It is easier for men to have access to the analyses. Women sometimes don't have the economic resources*.

(Community nurse, woman, focus group discussion)

*In the first months, I had expenses... but they were less because the charity and social services gave funds to me*.

(19 years old, woman, interview)

*If I had known before (about the free nature of the tuberculosis control program), I would have gone earlier*.

(22 years old, woman, interview)

Participants reported that being employed a considerable distance away from home and inflexible health establishment operating hours caused delays in tuberculosis test-seeking. This was reported to more heavily affect men who tended to work outside of their local community.

*Men do not leave their work. They allow more time to pass and the disease gets worse*.

(Clinic nurse, woman, focus group discussion)

#### 6. Tuberculosis treatment experiences

Overwhelmingly, the view expressed by the healthcare workers was that societal gender discrimination existed within the community and impacted on women's tuberculosis health experiences. They expressed the belief that discrimination against women with tuberculosis resulted in a lack of support from their families and sometimes led to abandonment and rejection by their spouses and family. They also believed that it was quite the reverse for men.

*We could say that of 100 percent, 70 percent of women are discriminated against and do not get much support from the family. The other 30 percent have husbands who try to help. They are aware of their diagnosis, their medication and accompany them to the health post*.

(Clinic nurse, woman, focus group discussion)

*We can see cases in which it is the husband that gets sick. He is told to rest. The woman assumes the responsibility of leader at home and also works. But in the case of the man, it is not the same. He does not offer support. In this case it would be economic support for the family but in the case of the woman it is totally different. She occupies the charge of the leader of the home. She makes a small business and takes responsibility for the family. The other factor is the psychological one. Here it is the women, who when ill, need emotional support, but the man does not offer this. In most cases he simply abandons them (the family). This is seen a lot nowadays*.

(Clinic nurse, woman, focus group discussion)

*In the case of a man, it is different. He has more support from his family, but the woman does not*.

(Community nurse, woman, focus group discussion)

*The rejection of a woman with tuberculosis by her family is more common. Her husband rejects her. She is afraid when she prepares dinner (referring to fear of infecting her husband). The woman on the other hand supports the man with tuberculosis. They help them through their treatment. The husband doesn't accompany her to receive her treatment*.

(Clinic technical assistant, woman, interview)

As reported in an earlier section, gender roles in this context meant that women were responsible for providing childcare. Combining treatment and family care was particularly burdensome for mothers who reported difficulty in arranging childcare whilst they visited the health post for treatment. Others who took their children to the health establishment often found it difficult because they had to leave their children outside during treatment. Some mothers said it would be preferable if health promoters came to their homes to give the medication, to ease difficulties with childcare.

*For those women with children, I think it is difficult to receive treatment. In the health post they didn't allow me to enter with my children and it was so difficult to leave them outside the health post*.

(34 years old, woman, interview)

*If the family is large and with children, it is difficult for the mother to seek medical help*.

(Clinic psychologist, man, interview)

It was frequently reported by women that tuberculosis was caused by overexertion at work. This, coupled with the stringent requirements of directly observed treatment, caused the few women who were in employment before diagnosis to give up their jobs. Many did not regain employment or return to work post-diagnosis.

*When I became ill, they told me that I should not work, that I should be recovering. I did not return to work because I feared I would relapse*.

(39 years old, woman, interview)

*It is easier for those that do not work (referring to coming to receive medication daily at the health post). For those that work it is difficult because of the work schedule*.

(34 years old, woman, interview)

A small number of patients reported that they had been able to arrange receipt of DOTS at the home of a health promoter, outside the health establishment's operating hours. Healthcare workers reported that this was informally arranged in order to facilitate patients' treatment whilst in employment. Men disproportionately used this service as they were more often employed.

#### 7. Tuberculosis-related stigma

Tuberculosis affected people were perceived negatively in this community, and women were found to be more affected by the socio-cultural consequences of tuberculosis, as shown below.

Participants described negative perceptions of tuberculosis patients on the part of the general population in Ventanilla. These negative perceptions resulted in rejection and prejudice for both men and women with the disease. Women, however, reported feeling the burden of tuberculosis stigma more heavily than men. For this reason, most chose to keep their diagnosis a secret from people outside their close family due to fear of rejection and isolation. Many women cited experiences of discrimination because of their diagnosis. This was in contrast to men who appeared unconcerned about the opinion of others with regards to their illness.

*If the neighbours had known that I was sick, they would have moved away from me, since people are ignorant. It happened with a neighbour when she had tuberculosis. The neighbours no longer accept water from her*.

(30 years old, woman, interview)

*My neighbours didn't know that I was sick. I had a normal life. I think that they didn't know and I didn't want them to know about it. I was always hiding when I went to take my tablets*...

(34 years old, woman, interview)

*At work, in the dining room, when people realised, they began to bring in their spoons and glasses and this made me feel bad*.

(34 years old, woman, interview)

*I had no interest in the opinion of others. The most important thing to me was the opinion of my family*.

(27 years old, man, interview)

All of the women interviewed reported having struggled with coming to terms with the diagnosis, the protracted treatment regimen and tuberculosis-related stigma.

*In the beginning, I took the news of the diagnosis badly. I thought that I would die*.

(34 years old, woman, interview)

*I knew that people would reject me. They worried that I would contaminate them. I have had some neighbours here in the past with tuberculosis. People see it like a disease that can't be cured. That is the worst thing, but it can be cured*.

(53 years old, woman, interview)

Women were particularly disturbed by excessive weight loss and their appearance when affected by tuberculosis.

*For the first 4 months, I was very thin; because of my appearance people got scared of me...When my sister was sick, they (her neighbours) discriminated against her. My sister was very thin and there was a girl that made fun of her and said that she had aborted a baby*.

(19 years old, woman, interview)

Female patients did not describe abandonment and complete isolation, however most had experienced subtle isolation as a result of their tuberculosis diagnosis.

*My husband threatened to leave me*.

(30 years old, woman, interview)

*Everything was not the same. I felt I was rejected in the family; they discriminated against me*.

(49 years old, woman, interview)

#### 8. Perceptions of tuberculosis services and personnel

Although there was a lot of praise for the tuberculosis program, participants identified several shortcomings. Inadequate healthcare infrastructure, particularly a lack of privacy within the health establishment whilst receiving DOTS care, was a complaint made mainly by women, but not men. Many complained about the structural layout and the cramped conditions in their local health establishment, which resulted in taking their medication in view of other patients.

*The place to take tablets is in the obstetrics area. Because people go for family planning, they always look at me. I get angry when they watch me*.

(19 years old, woman, interview)

*We are visible to other patients when we go to take our medication. The place is very small*.

(49 years old, woman, interview)

It was widely reported by patients and healthcare workers alike that direct observation of treatment was generally more distressing for women than men.

*I found it a little inconvenient. Everyday I had to go there... but I went there. Sometimes I felt inconvenienced but it was my health*.

(39 years old, woman, interview)

*She (the tuberculosis program staff) made me think I was irresponsible, but I think that it should not be this way, since I wanted to be cured. I felt bad because I had to take the drug so quickly*.

(34 years old, woman, interview)

I felt ashamed. It made me feel uncomfortable

(24 years old woman, interview)

It doesn't bother me to be supervised when taking the medicine. I just go to take my treatment and leave, nothing more!

(47 years old, man, interview)

Patients did, however, comment on the advantages of DOTS. They believed that without observation, some patients would not take their medication and put the community at greater risk of tuberculosis.

*When she is observing me, I ask myself why they don't give me the tablets to take home. But they want to observe if I take the tablets or not. Of course I feel a little bad...because it is like they are demanding that I take the tablets, but I remind myself that they are demanding me to get better, to be cured, but I still feel bad... I know people who have the disease but deny it. They do not take their tablets. I know they (the tuberculosis program staff) do this to protect all of us. They want to stop more disease in our community*.

(53 years old, woman, interview)

Both male and female patients reported that the tuberculosis program was not gender discriminatory.

*Between men and women, the attention and attitude is the same*.

(34 years old, woman, interview)

*There is no difference in the treatment of men and women. The staff at the health establishment treat us all the same*.

(31 years old, man, focus group discussion)

However, some community healthcare workers suggested that clinic-based personnel may be less likely to suspect tuberculosis in women, or may take their symptoms less seriously than those of men because they were more frequent attendees at the health establishment when compared to men.

*I think sometimes the woman may be dismissed because she is always at the health establishment. It is believed that if a man presents he must be in a bad way and actually have something serious wrong with him*.

(Community nurse, woman, focus group discussion)

A few women, particularly those with MDTR-TB, reported that they were deterred from seeking help because of what they perceived to be "scare-mongering" by healthcare staff. According to female patients, healthcare workers reinforced the message that if patients defaulted on treatment, they would get MDR-TB and probably die.

*I understand that the nurses use the "last chance tactic." They say, "Mrs X if you don't complete your treatment, if you don't attend everyday, if you relapse, we will no longer be able to help you. There is no further treatment, this is the last one"...Several times they have told us this. I sometimes think and I say, "Oh God, if I don't finish this treatment, if I relapse, they won't help me"... I think they tell us in this manner for us to get better, for the benefit of the patients, to complete our treatment... because they say if we miss one day of treatment, it will be worse for us*.

(53 years old, woman, interview)

## Discussion

Female and male tuberculosis patients and healthcare workers reported gender-specific differences and inequity in the health experience of patients affected by tuberculosis despite apparent equality in tuberculosis care provision. Five main contributory themes were derived from the categories explored: (a) gender roles; (b) low prioritization of women's health; (c) poverty; (d) service provision and DOTS; and (e) stigma (Table 2). Figure 1 shows a proposed conceptual framework to illustrate the interaction of these themes. This discussion is developed in the context of the sparse and geographically diverse literature concerning this important subject.

Gender roles and social norms contributed to women being considered inferior to men in this cultural setting. This resulted in a lower priority being given to women's versus men's health, particularly since men were primarily the financial decision-makers of the family [13]. This lower priority was evidenced by the need for women to obtain permission to spend money on their own healthcare and their lack of decision-making power, which prevented them from freely seeking appropriate healthcare for themselves [13]. It is important to emphasise that this lower health-seeking behaviour took place despite women being regular attendees at health clinics, where they often sought help for relatives and not for themselves. As with other studies, we found that the burden of caring for tuberculosis patients fell on women [18].

As reported in other studies, poverty amongst women due to limited work opportunities was found to reinforce these societal gender roles [19]. Stereotyped gender roles in this context, typically assigned primary responsibility for the household and childcare to women [20]. Although the National Tuberculosis Control Program is free at the point of care, there were hidden costs that included the entire cost of tests preceding the diagnosis of tuberculosis (other than sputum microscopy), travel, nutrition and lost earnings [21]. In the current study, women preferred to spend their limited resources on the needs of their family rather than on their healthcare. In some settings, it has been reported that women spend less on healthcare and are reluctant to consult modern health services, mainly because of the cost [6,19]. Furthermore, having tuberculosis has a detrimental effect on the economic lives of women [19]. Additionally the widely held belief amongst women that tuberculosis is caused by over-exertion at work caused many to give up their employment, potentially worsening poverty (see Fig. 1). Participants in this study reported that the inflexibility of health post operating hours contributed to diagnostic delay and prevented those who remained in employment from accessing DOTS therapy. This mainly affected men who were more likely to be employed [22].

We found that women experienced stigma and the socio-cultural consequences of tuberculosis more profoundly than men [13,20-22]. Rejection, abandonment and ostracism were found to be consequences of tuberculosis for some women with tuberculosis, while most women experienced more subtle isolation by community and family [19]. The stigma associated with tuberculosis places women in an economically and socially precarious position. It has been reported that single women with tuberculosis find it difficult to find partners [19]. As in other studies, in this context stigma was found to result in concealment of disease [7]. In some settings women report a higher burden of psychological suffering as a result of tuberculosis [6,13] and hence may benefit from psychological services as part of their care package.

Despite equal tuberculosis care provision for men and women, women were found to be more affected by deficiencies in the healthcare infrastructure and the attitudes of staff [7], potentially resulting in poorer health outcomes for women (see Fig. 1). The healthcare infrastructure was of concern to women because the layout of most health establishments limited privacy at the point of delivery of DOTS therapy. Some health posts responded to this by creating separate outhouses and entrances for the tuberculosis program. Previous research has suggested that supervised treatment is insensitive to female needs and a randomised controlled study in South Africa found greater treatment success amongst women who received self-supervised treatment, compared to those who received DOTS [23]. Treatment supervision also proved difficult for mothers with children, where arranging childcare was a barrier to accessing DOTS.

The attitude of healthcare professionals towards patients was steered by an equality principle and was generally found not to be gender discriminatory, in contrast to a previous study in Peru [24]. It was suggested by Thorson (2004) that professionals' attitudes towards patients should be steered by the equity principle, because when an equality principle is applied to a situation in which persons are unequal, the result may be gender blindness [19]. Hence differential treatment is suggested for individual patients according to their needs [19]. Additionally the practice of healthcare workers over-emphasising the potential end result of death as a consequence of non-compliance with tuberculosis treatment deterred women with MDR-TB from seeking help.

There are some limitations to the research methods and analysis used. It has been found that interviewees are affected by interviewer characteristics such as appearance and race [15]. In order to address this, interviews were conducted jointly by the lead investigator and a community healthcare worker of Peruvian origin. The grounded theory approach to analysis requires the person carrying out the analysis to put aside all previous knowledge of relevant theories and concepts in order to solely rely on the data collected [15]. This was facilitated by "bracketing," through which the researcher remained aware of her own preconceived ideas and made attempts to prevent these from over-riding the participants' accounts of their own reality [15]. Although the sample size was small, data collection activities were carried out to the point of saturation, when no new ideas emerged.

Reporting and recall bias may also have occurred. Attempts were made to minimize these by probing and reconfirming earlier responses. Due to the use of purposive sampling, selection bias was a possibility. This was addressed through the triangulation of sources and methods, whereby accounts about the health experiences of male and female tuberculosis patients were obtained from different sources (patients and healthcare workers) using two different methods (semi-structured interviews and focus groups). Finally, although there were more female health worker participants than male, this was proportionate to their representation within the tuberculosis program at health establishments in Ventanilla.

Future research is recommended to examine whether the gender difference in tuberculosis prevalence is due to biological differences or gender-related social prejudice impacting on health-seeking behaviour. Prevalence studies may help to clarify this. Further studies to assess the magnitude of gender barriers to controlling tuberculosis are called for, including adolescents and children, as are interventional studies to negate these barriers.

## Conclusions

Our findings have implications for tuberculosis control in Peru and other resource-poor settings with a high tuberculosis burden. We discovered gender-related barriers to tuberculosis control despite ostensibly equal tuberculosis care provision. This was found to be reflective of socio-cultural norms that place a lower priority on the health of women compared to men, and because women who attended the health establishments were usually balancing multiple responsibilities including care for children and other family members. Women were not expected to seek healthcare for themselves.

Discussion of a gender specific approach within tuberculosis programmes is needed in order to address these barriers to tuberculosis control. Additionally, public education initiatives should work to reduce the fear and stigma associated with tuberculosis. The greatest opportunities, however, appear to be in social education of the population and provision of job opportunities for women in order to promote gender equity and to break the cycle of gender-related social prejudice and poverty.

## Competing interests

The authors declare that they have no competing interests.

## Authors' contributions

DAO conceived and led all aspects of this research with mentorship from CAE. The field work was led by RM supported by MH, JA, JF, RS, BV, and EV. CMF was responsible for the qualitative and quantitative research which formed the foundation of this study and contributed to the study design. CDA contributed to the stigma-related aspects of the research. AMB led the qualitative methods and interpretation. All authors contributed to the interpretation of the qualitative data and the manuscript preparation.

## Pre-publication history

The pre-publication history for this paper can be accessed here:

http://www.biomedcentral.com/1471-2458/10/381/prepub
